# Identification of *Ophiocordyceps sinensis* and Its Artificially Cultured *Ophiocordyceps* Mycelia by Ultra-Performance Liquid Chromatography/Orbitrap Fusion Mass Spectrometry and Chemometrics

**DOI:** 10.3390/molecules23051013

**Published:** 2018-04-26

**Authors:** Ping Zhang, Saina Li, Juan Li, Feng Wei, Xianlong Cheng, Guifeng Zhang, Shuangcheng Ma, Bin Liu

**Affiliations:** 1 School of Chinese Material Medica, Beijing University of Chinese Medicine, No. 11Beisanhuan east Road, Beijing 100029, China;zping0227@sina.com; 2 Research and Inspection Center of Traditional Chinese Medicine and Ethnomedicine, National Institutes for Food and Drug Control, State Food and Drug Administration, No. 2 TiantanXili, Beijing 100050, China; weifeng@nifdc.org.cn (F.W.); lncxl@sina.com (X.C.); 3 State Key Laboratory of Biochemical Engineering, Institute of Process Engineering, Chinese Academy of Sciences, No. 1 Second north Road of zhongguancun, Beijing 100190, China;seine2015@163.com (S.L.); gfzhang@ipe.ac.cn (G.Z.); 4 Institute of Microbiology, Chinese Academy of Sciences, No. 1 West Beichen Road, Beijing 100101, China; 18612037125@126.com

**Keywords:** *Ophiocordyceps sinensis*, ultra-performance liquid chromatography/Orbitrap Fusion mass spectrometry, chemometrics, fungi marker peptide, quality control

## Abstract

Since the cost of *Ophiocordyceps sinensis*, an important fungal drug used in Chinese medicine, has increased dramatically, and the counterfeits may have adverse health effects, a rapid and precise marker using the peptide mass spectrometry identification system could significantly enhance the regulatory capacity. In this study, we determined the marker peptides in the digested mixtures of fungal proteins in wild *O. sinensis* fruiting bodies and various commercially available mycelium fermented powders using ultra-performance liquid chromatography/Orbitrap Fusion mass spectrometry coupled with chemometrics. The results indicated the following marker peptides: TLLEAIDSIEPPK (*m*/*z* 713.39) was identified in the wild *O. sinensis* fruiting body, AVLSDAITLVR (*m*/*z* 579.34) was detected in the fermented *O. sinensis* mycelium powder, FAELLEK (*m*/*z* 849.47) was found in the fermented *Ophiocordyceps* mycelium powder, LESVVTSFTK (*m*/*z* 555.80) was discovered in the artificial *Ophiocordyceps* mycelium powder, and VPSSAVLR (*m*/*z* 414.75) was observed in *O. mortierella* mycelium powder. In order to verify the specificity and applicability of the method, the five marker peptides were synthesized and tested on all samples. All in all, to the best of our knowledge, this is the first time that mass spectrometry has been employed to detect the marker peptides of *O.sinensis* and its related products.

## 1. Introduction

Chongcao (the sexual stage of the *Ophiocordyceps sinensis*) is an important traditional fungal drug that has been commonly used for hundreds of years as a tonic and/or drug. However, its safety was questioned, because the wild *Ophiocordyceps sinensis* was reported to contain a high amount of arsenic likely due to soil contamination [1]. Then *Ophiocordyceps sinensis* had the clinical effect of tonifying the kidney and replenishing lung, stanching bleeding, and resolving phlegm. It could be used to treat 21 ailments and also be a potential adjuvant chemotherapeutic agent in non-small cell lung cancer, liver cancer, and breast therapy [2,3]. Despite the hazardous effects to the humanhealth, chongcao possesses manyanti-tumor and antioxidant activities, as well asthe capacity to modulate the immune system and treat fatigue, night sweating, hyposexuality, hyperglycemia, liver disease, and heart disease [4,5,6,7,8,9]. In recent years, due tothe limited natural resources and simultaneously increasing demand, thecost of *O. sinensis* has increased dramatically. In spite of the increasing price, its manufacture and sales were strictly regulated in 2016 by the China Food and Drug Administration (CFDA), because its natural fruiting bodies usually contain high amounts of arsenic, which is an environmental pollutant and could decrease neuronal migration, as well as cellular maturation, and it inhibits the proliferation of neural progenitor cells [1,10]. Considering the safe clinical use and the discrepancy between need and availability, other *Ophiocordyceps*-related fungi and the conidial forms of the artificially cultured *O. sinensis* fermentation mycelia have been used as substitutes in Chinese medicine and healthy food [11,12].

From the numerous species that have been reportedly isolated from *O. sinensis* [13,14], it is widely accepted among researchers that *Hirsutella sinensis* is a unique anamorph of *O. sinensis* [11,15,16,17], while other species such as *Paecilomyces hepialid*, *Gliocladium roseum*, and *Tolypocladium sinensis* represent endoparasitic fungi commonly found in natural *O. sinensis* [18,19]. Currently, four species isolated from *O. sinensis*, namely, fermented *O. sinensis* mycelium powder (*Hirsutella sinensis* species), fermented *Ophiocordyceps* mycelium powder (*Paecilomyces hepialid* species), artificial *Ophiocordyceps* mycelium powder (*Gliocladium roseum* species), and *O. mortierella* mycelium powder (*Tolypocladium sinensis* species), all of which are usually confused with the *O. sinensis* name, have been successfully cultured. Four more standardized mycelia fermentation products of *Ophiocordyceps* have been produced [20,21,22,23,24] and are widely employed as Chinese medical material in preparations in China [25].

Since different *Ophiocordyceps* species may have different health effects, authentication of *O. sinensis*-related products is essential in order to ensure safeuse and efficacy. Traditionally, *O. sinensis* is identified through morphological description, microscopic identification, or chemical composition assay [23,26,27,28,29]. However, since these methods lack objective standards or a specific component index, it is difficult to effectively identify and distinguish *O. sinensis* from various cultured *O.* mycelia [30,31,32,33,34]. Furthermore, although the polymerase chain reaction (PCR) has been successfully applied in the identification of *Ophiocordyceps sinensis* (*O.S.*) fruiting bodies [14,15,35], it cannot be used for cultured *Ophiocordyceps* mycelia, because the integrity of the DNA genome is compromised during the drying process [18,36,37].

Since species identification is an important and necessary procedure to control the quality and standardization of herbal medicines, it is crucial to develop methods to authenticate *O. sinensis* and the four aforementioned cultured *O.* mycelia. One approach to that could be the detection of the fungal proteins in the species. The fungal protein is a special protein, the amino acid sequence of which is different in the different fungi, thereby making it an important factor in the identification of species [38,39]. Moreover, these proteins are among the bioactive components in *O.S.* and, to the best of our knowledge, have rarely been studied and reported [40]. Biological mass spectrometry has been developed as an efficient method for proteomic analysis that exhibits superior mass accuracy and ultra-high resolution, and employs a segmented quadruple mass filter with improved selectivity and ion transmissibility [38,39,41,42,43,44,45,46]. However, for a specific genus, there is only limited data available to characterize the fungal protein. One example is the marker peptide in the digested mixture of *O. sinensis*-related products.

In this study, we first determinedthe digested mixture peptides by ultra-performance liquid chromatography/Orbitrap Fusion mass-spectrometry (UPLC/MS/MS). Then, the marker peptide ion was detected using chemometrics, and the marker peptide sequence was confirmed by comparisonwith the database for the identification of *O. sinensis* and relevant cultured *Ophiocordyceps* mycelia. The results demonstrated that this method could be used to authenticate not only wild *O. sinensis* and its related cultured *Ophiocordyceps* mycelia powder but also the mixed commercial products. Moreover, the work presented herein is, to the best of our knowledge, the first extensive study on the authentication of *O. sinensis* and cultured *Ophiocordyceps* mycelia using mass spectrometry and chemometrics, thereby providing a powerful quality control tool.

## 2. Results

### 2.1. Size-Exclusion Chromatographic Analysis ofFungal Proteinsand Their Tryptic Digest Mixtures

**Figure 1:**
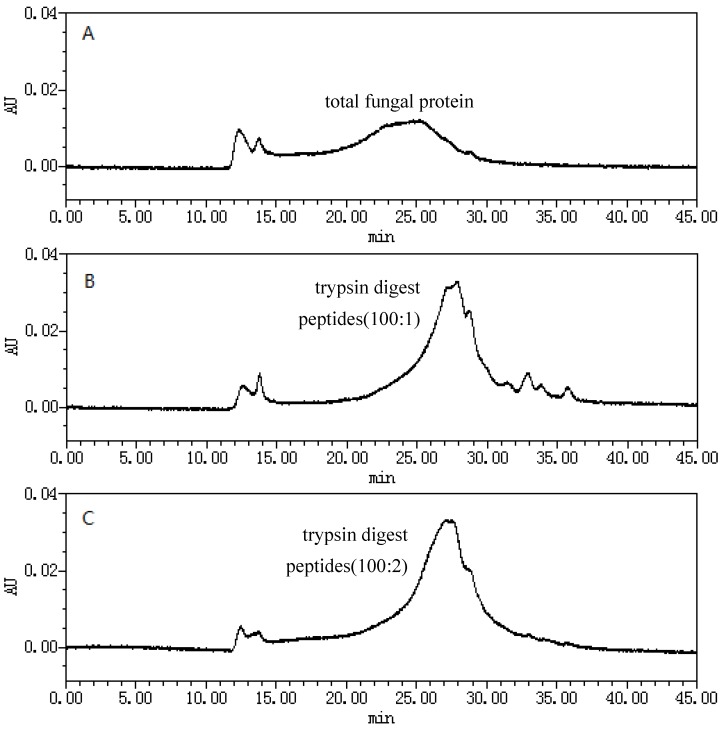

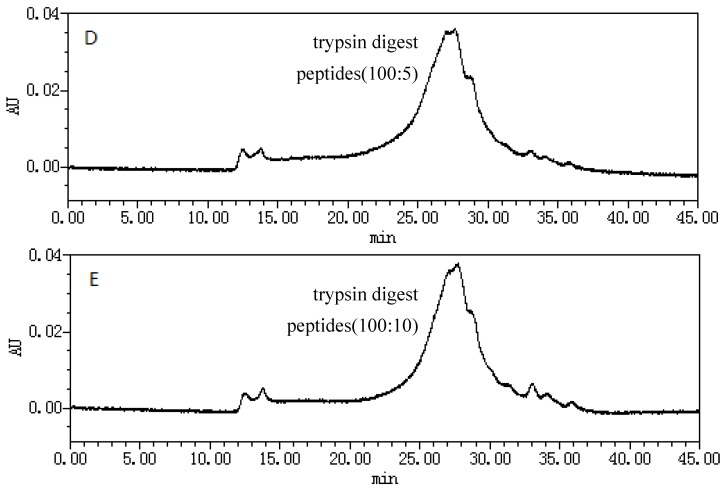
The gelfiltration chromatograms of (**A**) fungal protein from fermented *O. sinensis* mycelia powder and (**B**–**E**) therespective digest peptidesincubated at 37 °C for 18 h with sample-to-trypsin ratios of (**B**) 100:1; (**C**) 100:2; (**D**) 100:5; and (**E**) 100:10.

Figure 1 shows the size-exclusion chromatograms (SEC) of fungal proteins in fermented *O. sinensis* mycelia and the sample aliquots withdrawn during the digestion process. It was found that the fungal proteins had a wide molecular weight range (Figure 1A). When the amount of trypsin was increased, the peak intensities arising from the digest mixture gradually increased, indicating that more peptides in the mixture were degraded (Figure 1B–D). Once the sample-to-trypsin ratio exceeded 100:10, no significant changes in the peak intensity in the elution profile were observed (Figure 1E). The molecular-weight ranges of the fungal proteins and the digest mixtures incubated at 37 °C for 18 h were assessed against a series of protein standards, namely immunoglobulin G (molecular weight (*M_W_* = 150 kDa), bovine albumin (*M_W_* = 68 kDa), globular actin (*M_W_* = 42 kDa), trypsinogen (*M_W_* = 24 kDa), lysozyme (*M_W_* = 14 kDa), and bovine insulin (*M_W_* = 6 kDa), which were analyzed by SEC under the same conditions. The results revealed that the molecular weights of the fungal proteins ranged from 42 to 14 kDa, whereas the molecular weights of the peptides in the digest mixture were <5 kDa, which is consistent with the theoretical molecular weight range of peptides resulting from the digestion of fungal proteins [47].

### 2.2. Multivariate Data Analysis

**Figure 2:**
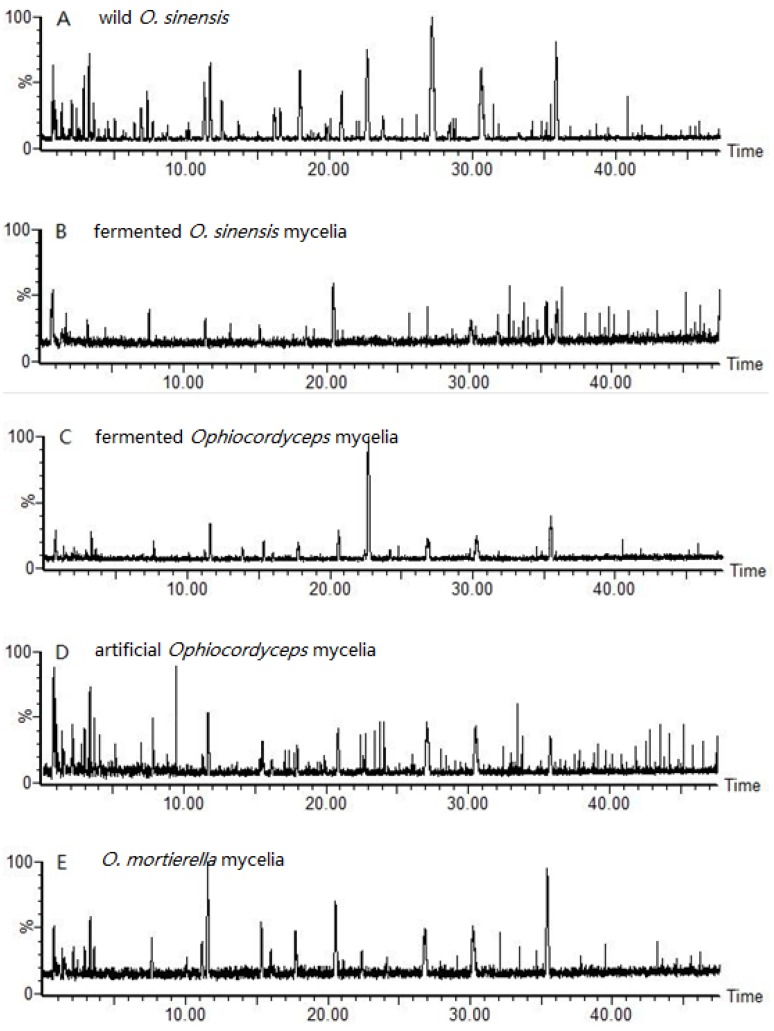
The positive-ion base-peak-intensity chromatograms of the digest peptides of: (**A**) wild *O. sinensis* fruiting body; (**B**) fermented *O. sinensis* mycelia powder; (**C**) fermented *Ophiocordyceps* mycelia powder; (**D**) artificial *Ophiocordyceps* mycelia powder; and (**E**) *O. mortierella* mycelia powder.

**Figure 3:**
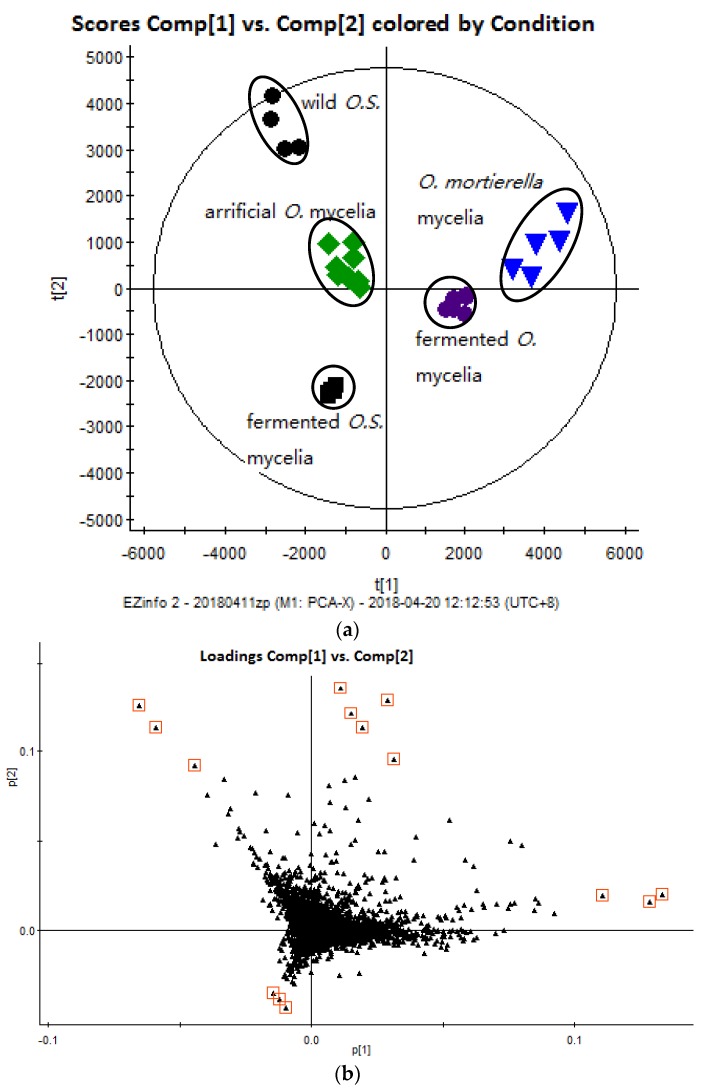
(**a**) The principal component analysis score plot of (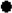): wild *O. sinensis* fruiting body, (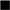): fermented *O.S.* mycelia powder, (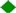): artificial *Ophiocordyceps* mycelia powder, (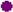): fermented *Ophiocordyceps* mycelia powder, (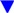): *O. mortierella* mycelia powder, and (**b**) the loading plot of wild *O. sinensis* and four cultured *Ophiocordyceps *mycelia.

The total ion chromatograms (TICs) of the wild *O. sinensis* and four cultured *Ophiocordyceps* mycelia fungal proteins digested at 37 °C for 18 h, over the 350–1550 *m*/*z* scan range, are displayed in Figure 2. The results show that the marker peptides of each mycelium were concealed by a large number of tryptic peptides that were indistinguishable in the TICs at higher concentrations due to the homologies of the fungal proteins. As a consequence, the different types of mycelia were difficult to detect simply by visual inspection of their chromatograms, and further sample profiling of the digest mixtures was performed using multivariate statistical software tools. In this study, the three-dimensional (3-D) ultra-performance liquid chromatography/Orbitrap Fusion mass-spectrometry (UPLC/MS/MS) data were first converted into a 2-D matrix containing exact-mass/retention-time (EMRT) pairs using the Progenesis QI for Proteomics, which is the application manager for Progenesis QI^TM^. The data set was visualized using unsupervised principal component analysis (PCA) in order to check for outliers and classification trends among the mycelia. Preliminary PCA was performed on all observations using pareto-scaled variables. The final PCA score plot revealed that five different types of mycelia clusters formed, all of which lay inside the Hotelling T2 (0.95) ellipse (Figure 3a). In the PCA scores plot, the fermented *Ophiocordyceps* mycelia powder and *O.* mortierella mycelia powder lay close to each other but were located much further away from the wild O. sinensis, the fermented *O.S*. mycelia powder, and the artificial *Ophiocordyceps* mycelia powder.

### 2.3. Identification of Marker Peptides in Digested Mixtures

The results from this study demonstrate that it is possible to isolate and identify marker peptides that play important roles in the authentication of various *Ophiocordyceps* mycelia. The loading plot from the PCA, based on the UPLC/MS data, is shown in Figure 3b. The ions that correspond to the EMRT pairs of 41.17–713.3967, 35.03–579.3498, 22.68–849.4741, 26.05–555.8072, and 13.60–414.7517 were chosen as marker peptides for each sample. The amino acid sequence of the marker peptides was determined using Mascot v2.5.1. Mascot was calibrated by searching the data on *O. sinensis*, *Hirsutella sinensis*, *Paecilomyces hepialid*, *Gliocladium roseum*, and *Tolypocladium sinensis*, which was obtained from the Universal Protein (UniProt) database. The Mascot search was performed with a fragment ion mass tolerance of 0.60 Da and a parent ion tolerance of 15.0 ppm. Carbamidomethylated iodoacetamide (IAM) was specified in Mascot as a fixed modification, while oxidized dithiothreitol (DTT) was specified as a variable modification.

**Figure 4:**
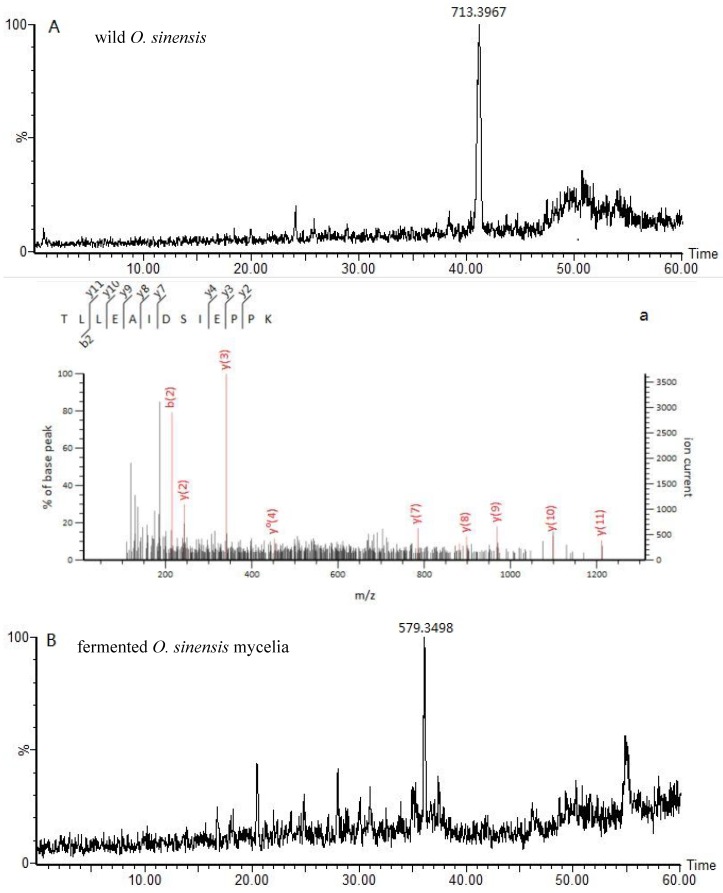

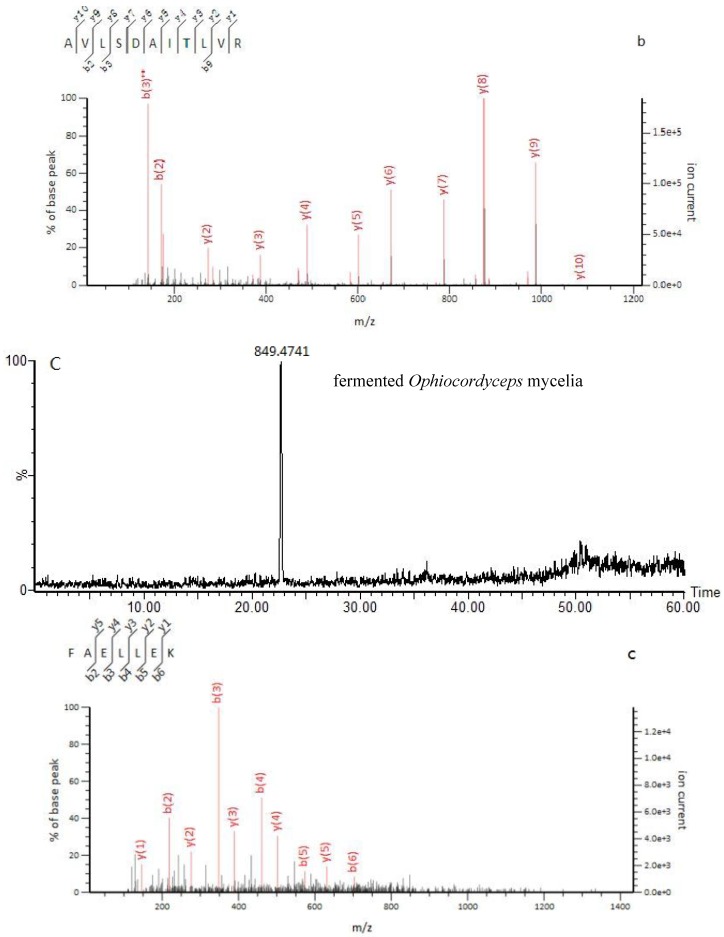

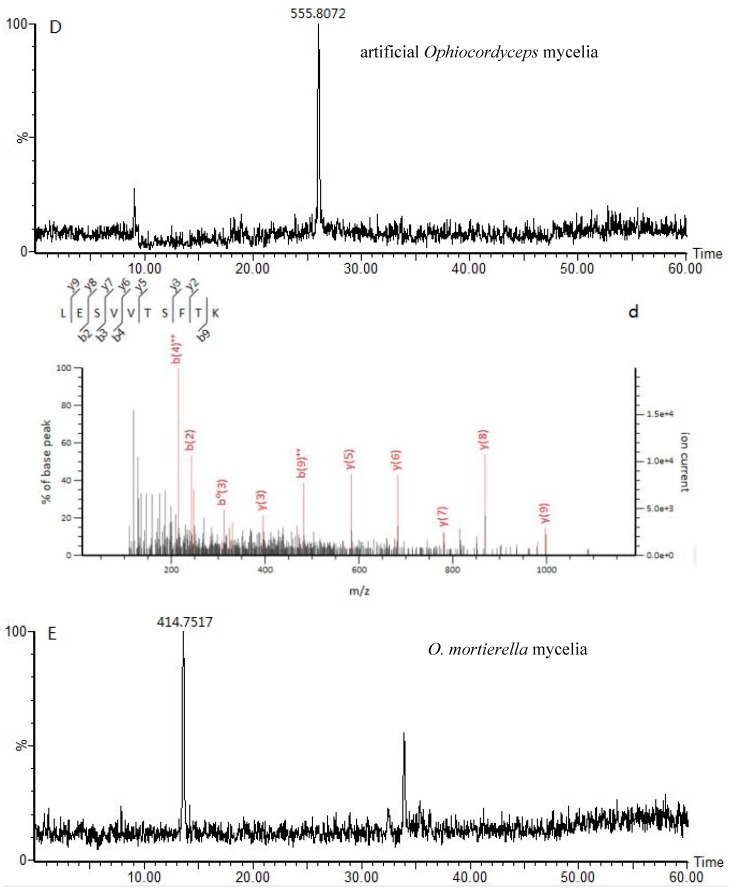

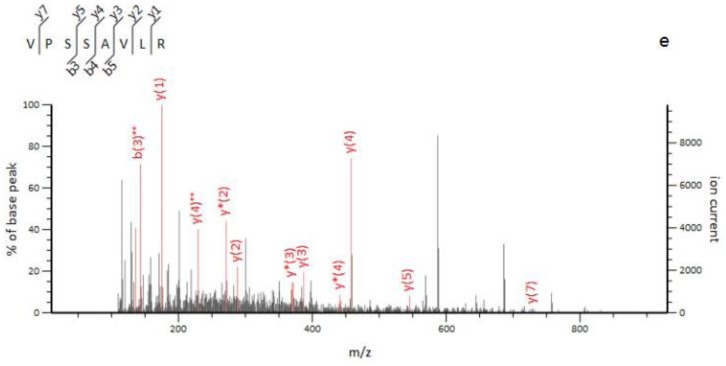
The selected ion-monitoring chromatograms of marker peptides in (**A**) wild *O. sinensis* fruiting body, *m*/*z* 713.39, a doubly charged TLLEAIDSIEPPK fragment ion; (**B**) fermented *O. sinensis* mycelia powder, *m*/*z* 579.34, a doubly charged AVLSDAITLVR fragment ion; (**C**) fermented *Ophiocordyceps* mycelia powder, *m*/*z* 849.47, a singly charged FAELLEK fragment ion; (**D**) artificial *Ophiocordyceps* mycelia powder, *m*/*z* 555.80, a doubly charged LESVVTSFTK fragment ion; and (**E**) *O. mortierella* mycelia powder, *m*/*z* 414.75, a doubly charged VPSSAVLR fragment ion. Correspondingly, the fragment ion mass spectrogram of marker peptides in (**a**) wild *O. sinensis* fruiting body; (**b**) fermented *O. sinensis* mycelia powder; (**c**) fermented *Ophiocordyceps* mycelia powder; (**d**) artificial *Ophiocordyceps* mycelia powder; and (**e**) *O. mortierella* mycelia powder.

The results of the study showed that the ion with *m*/*z* = 713.3967 was only found in the spectrum of the digested mixture of the wild *O.S.* fruiting body (Figure 4A). Moreover, extracted-ion mass spectrometry (MS^E^) revealed that this ion was doubly charged and that the MS/MS spectrum indicated that it corresponded to the TLLEAIDSIEPPK amino acid sequence. The partial LEAIDSIEPPK amino acid sequence was derived from the observed single charged y(11) *m*/*z* 1211.6518 ion, while the partial TL amino acid sequence was confirmed by the b(2) *m*/*z* 215.1390 ion. In contrast, the ion with *m*/*z* = 579.3498 was only detected in the spectrum of the digested mixture of the fermented *O.S*. mycelia powder (Figure 4B). Similarly, the respective MS^E^ spectrum revealed that this ion was doubly charged, while the MS/MS spectrum indicated that it corresponded to the AVLSDAITLV amino acid sequence. The partial AVL amino acid sequence was derived from the observed doubly charged b^++^(3) *m*/*z* 142.6021 ion, while the partial SDAITLVR amino acid sequence was confirmed by the y(8) *m*/*z* 874.4993 ion. In addition, the ion with *m*/*z* =849.4741 was only found in the spectrum of the digested mixture of the fermented *Ophiocordyceps* mycelia powder (Figure 4C). The MS^E^ spectrum revealed that this ion was single charged, and that its MS/MS spectrum indicated that it corresponded to the FAELLEK amino acid sequence. The partial FAE amino acid sequence was derived from the observed single charged b(3) *m*/*z* 348.1554 ion, while the partial LLEK amino acid sequence was confirmed by the y(4) *m*/*z* 502.3235 ion. Moreover, the ion with *m*/*z* = 555.8072 was only detected in the spectrum of the digested mixture of the artificial *Ophiocordyceps* mycelia powder (Figure 4D). The MS^E^ spectrum revealed that this ion was doubly charged, and that its MS/MS spectrum indicated that it corresponded to the LESVVTSFTK amino acid sequence. The partial LESV amino acid sequence was derived from the observed doubly charged b^++^(4) *m*/*z* 215.1208 ion, while the partial VTSFTK amino acid sequence was confirmed by the y(6) *m*/*z* 682.3770 ion. Lastly, the ion with *m*/*z* = 414.7517 was only found in the spectrum of the digested mixture of the *O. mortierella* mycelia powder (Figure 4E). The corresponding MS^E^ spectrum revealed that this ion was doubly charged, and that its MS/MS spectrum indicated that it corresponded to the VPSSAVLR amino acid sequence. The partial VPS amino acid sequence was derived from the observed doubly charged b^++^(3) *m*/*z* 142.5839 ion, while the partial SAVLR amino acid sequence was confirmed by the y(5) *m*/*z* 545.3406 ion.

The amino acid sequences of the marker peptides of each *Ophiocordyceps* species were aligned using the Basic Local Alignment Search Tool (BLAST) of the UniProt database in order to identify the original protein types. The results were as follows: translation elongation factor 1-α (gi:A4U9H1), belonging to *Ophiocordyceps brunneipunctata* (Table 1) and recognized by Mascot matching as a precursor of the tryptic peptide TLLEAIDSIEPPK (*m*/*z* 713.3967), was chosen as a marker of the wild *O. sinensis* fruiting body; linoleate diol synthase (gi: T5AC53), belonging to *Hirsutella sinensis* and recognized by Mascot matching as a precursor of tryptic peptide AVLSDAITLVR (*m*/*z* 579.3498), was chosen as a marker for unambiguous identification of the fermented *O. sinensis* mycelia powder; the adenosine triphosphate (ATP) synthase subunit α (gi: A0A0B7JUZ6), belonging to *Bionectria ochroleuca* (*Gliocladium roseum*) and recognized by Mascot matching as a precursor of tryptic peptide LESVVTSFTK (*m*/*z* 555.8072), was chosen as a marker of the artificial *Ophiocordyceps* mycelia powder(*Gliocladium roseum* species). However, the two ions at *m*/*z* 849.4741 and 414.7517 were not assigned to any protein by the Mascot matching. The selected ion monitoring chromatograms of the marker peptides and the corresponding spectra (MS^E^) are shown in Figure 4. In most cases, the amino acid sequences were recognized to belong to specific proteins of the analyzed species, while in other cases, the peptide was not assigned to any protein, with the engine indicating only partial matching (in brackets, Table 1).

## 3. Discussion

Due to its apparent beneficial clinical and health effects, *O. sinensis* has been employed in China as a highly valued traditional Chinese medicine for centuries. Recently, it has become increasingly popular and is now widely used, especially by elderly and unhealthy people in China and abroad, as a dietary supplement or natural remedy [25,48]. However, the market price for *O. sinensis* has increased remarkably due to insufficient resources and growing demand. Moreover, other *Ophiocordyceps*-related fungi and the conidial form of the artificially cultured *O. sinensis* fermentation mycelia have also been used as substitutes in Chinese medicine and healthy foods [11,12], thereby causing confusion in the market management and challenging the safe use of *O. sinensis*. Therefore, it is crucial to develop a reliable and practical method to differentiate *O. sinensis* from its substitutes, especially the cultured mycelia.

In this study, the marker peptides in the digest mixtures of fungal proteins were determined by UPLC/MS/MS coupled with chemometrics using wild *O.S.* fruiting bodies and several commercially available mycelium fermented powders. Moreover, the marker peptides were detected, and the amino acid sequences of the marker peptides were identified. The obtained results showed that the marker peptides could provide accurate species identification for the *Ophiocordyceps* samples by biological mass spectrometry. To the best of our knowledge, the first extensive study on the authentication of *O. sinensis* and revelent-cultured *Ophiocordyceps* mycelia by biological mass spectrometry combined with chemometrics, thereby provided a powerful quality control tool.

Previous studies report different macroscopic and microscopic methods that can be used to identify *O. sinensis*-related products [23,26,27,28,29]. Nevertheless, the probability of the accurate identification of the species level was not the same for the different species [32,49,50,51,52]. Most studies focused on morphological characterizations, microscopy studies, determination of the chemical composition, and PCR amplifications. However, none of them analyzed the specific fungal protein or, more specifically, the marker peptide that identifies the species level of *O. sinensis*. 

The fungal protein is a special protein that is differentiated by the type of fungus [40]. One efficient method for proteomic analysis is biological mass spectrometry [39,53,54]. In recent years, many studies have focused on the fungal proteins. Two dimensional electrophoresis (2-DE) and sodium dodecyl sulfate-polyacrylamide gel electrophoresis (SDS-PAGE) was used to examine and identify *O. sinensis* [40,55]. Then, five proteins were identified using MALDI-TOF-TOF/MS. Based on the proteomic profile of *O. sinensis*, 2-DE identification pattern was provided, and this approach was a foundation for intensive study of *O. sinensis* proteins. Another isobaric tag for relative and absolute quantification (i TRAQ)-coupled two-dimensional liquid chromatography tandem mass spectrometry(2D LC-MS/MS) proteomics approach was used to analyze the protein profiles of samples of the larva and various development stages of Chinese *Cordyceps*. This bioinformatics analysis revealed that i TRAQ-coupled 2D LC-MS/MS was a unique method for identifying protein groups of Chinese *Cordyceps* at different development stages [56]. None of these methods can effectively identify and distinguish *Ophiocordyceps sinensis* and its revelent-fermented *Ophiocordyceps* mycelia. In this study, we employed this approach to identify the marker peptides of the specific fungal proteins in wild *O. sinensis* and four revelent-fermented *Ophiocordyceps* mycelia powders. Then, as a result, TLLEAIDSIEPPK (*m*/*z* 713.39) was detected in wild *O. sinensis* fruiting bodies, which was matched to protein of translation elongation factor 1-α, belonging to *Ophiocordyceps brunneipunctata.* AVLSDAITLVR (*m*/*z* 579.34) was discovered in the fermented *O. sinensis* mycelium powder, which was matched to protein of linoleate diol synthase, belonging to *Hirsutella sinensis.* FAELLEK (*m*/*z* 849.47) was found in fermented *O.* mycelium powder, which was not matched to any protein. LESVVTSFTK (*m*/*z* 555.80) was identified in artificial *O.* mycelium powder, which was matched to protein of ATP synthase subunit α, belonging to *Gliocladium roseum*. VPSSAVLR (*m*/*z* 414.75) was detected in *O. mortierella* mycelium powder, which was not matched to any protein. All in all, three marker peptides were matched to the corresponding species; two marker peptides were not matched to corresponding species, but they were specific peptides. 

In order to verify their specificity, all marker peptides were synthesized and tested on the samples. The results from the measurements of the aforementioned five samples revealed the following information on the marker peptides: TLLEAIDSIEPPK was only present in wild *O. sinensis*, AVLSDAITLVR was only detected in fermented *O. sinensis* mycelium powder, FAELLEK was only observed in fermented *Ophiocordyceps* mycelium powder, LESVVTSFTK was only present in artificial *O.* mycelium powder, and VPSSAVLR was only found in *O. mortierella* mycelium powder. Previous studies revealed the proteins from 26 different producing areas were obviously different in the numbers and abundance of protein spots of protein profiles, and this showed certain association with producing areas [40]. Another report revealed five proteins of *O. sinensis* were identified in 2-DE, but the specific protein was not reported [55]. Isobaric tags for relative and absolute quantification (i TRAQ)-coupled two-dimensional liquid chromatography tandem mass spectrometry (2D LC-MS/MS) proteomics approach was used to analyze the protein profiles of samples of the larva and various development stages of Chinese *Cordyceps*. The results indicated that protein composition of mummified larva, sclerotium, and stroma were significantly different from commercial *cordyceps* [56]. These were the results of studying the producing area and various development stages of *O. sinensis*. Few study results of the differential proteins of *O. sinensis* and various cultured *Ophiocordyceps* mycelia were reported. In our study, the specific marker peptides were found by chemomatrics first and identified the sequence using MASCOT. Thus, we could examine the marker peptides to identify the *O. sinensis* and revelent-fermented *Ophiocordyceps* mycelia products. 

The method developed in this study could be applied not only to qualitatively identify the *O. sinensis*-related species, but also to quantitatively determine the contents of the marker peptides to control the qualityof the *Ophiocordyceps* related products. Moreover, the biological mass spectrometry method is also suitable for the identification of Chinese medicinal materials derived from animals, especially processed animal medicinal materials such as *Cicadae periostracum*, processed *Manis squama*, etc. However, because Chinese herbal medicine contains many complex ingredients, the extraction of high-purity and high-quality protein components is a key requirement of this method and its application to traditional Chinese medicine (TCM).

## 4. Materials and Methods

### 4.1. Materials and Reagents

Polyacrylamide and Coomassie Brilliant Blue were purchased from Sigma-Aldrich (St. Louis, MO, USA). Acetic acid was purchased from Beijing Chemical Reagent Co. (Beijing, China), while dithiothreitol (DTT), iodoacetamide (IAM), and trifluoroacetic acid (TFA) were purchased from Sigma-Aldrich (St. Louis, MO, USA). The reagents used were either of chemical or analytical reagent grade. Ammonium hydrogen carbonate (analytical reagent grade) was purchased from Beijing Chemical Reagent Co. (Beijing, China); formic acid was obtained from Sigma-Aldrich (St. Louis, MO, USA); and acetonitrile (HPLC grade) was purchased from Fisher Scientific (Fair Lawn, NJ, USA). The ultra-high-purity water was prepared using a Milli-Q water purification system (Millipore Corporation, Bedford, MA, USA). Trypsin (sequencing grade) was obtained from Pierce (Thermo Scientific, Waltham, MA, USA). The syringe filters (diameter: 0.22 µm) were purchased from Millipore (Billerica, MA, USA). Four wild *O.S.* samples were collected from the Tibetan province in China. The sources were identified by Chief Pharmacist Zhang nan-ping of National Institutes for Food and Drug Control (NIFDC) in China. Five fermented *O. sinensis* mycelia samples were provided by Hangzhou Sino-US Huadong Pharmaceutical Co., Ltd. (Hangzhou, China), and six fermented *Ophiocordyceps* mycelia samples were obtained from Jiangxi Jiminkexin Pharmaceutical Co. Ltd. (Nanchang, China). Eight artificial *Ophiocordyceps* mycelia samples were provided by Hebei Chang Tian Pharmaceutical Co., Ltd. (Shijiazhuang, China), and five *O. mortierella* mycelia samples were obtained from Zhejiang Changxing Pharmaceutical Co. Ltd. (Hangzhou, China) (Table 2). These strains of four fermented *Ophiocordyceps* mycelia were identified by Institute of Microbiology, Chinese Academy of Sciences in China.

### 4.2. Extraction of Ophiocordyceps Fungal Proteins

First, deionized water (0.5 mL) was added to a collected sample (10 mg) in a 1.5-mL microfuge tube. The sample was mixed, centrifuged for 5 min at 20,000× *g*, and the supernatant was completely removed. Subsequently, a lysis buffer (30 µL) and silica powder (Φ 0.5 mm) were added to the tube, and its contents were ground repeatedly with a plastic pestle for 2 min using twisting motions. Next, lysis buffer (150 µL) was added, and the sample was ground again for 30 s with the same pestle. The tube was centrifuged for 5 min at 14,000× *g*, the supernatant was collected in a new tube, and another aliquot of a lysis buffer (150 µL) was added to the prime tube. The sample was ground again for 30 s with the same pestle, and the tube was centrifuged for 5 min at 14,000× *g*. The supernatant was collected and transferred to a Millipore 3K ultrafiltration spin column placed in a 2-mL collection tube. The spin column was centrifuged for 25 min at 12,000× *g*, after which the filtrate was discarded and NH_4_HCO_3_ (300 µL, 0.05 mol/L, pH 8.0) was added. The spin column was centrifuged for 25 min at 12,000× *g*, and the filtrate was discarded again. This step was repeated twice before the spin column was inverted into a new2-mL collection tube and centrifuged for 5 min at 14,000× *g*. Finally, another aliquot of NH_4_HCO_3_ (300 µL, 0.05 mol/L, pH 8.0) was added, and the sample was diluted to a concentration of 1 mg/mL [57,58,59].

### 4.3. Purification and Tryptic Digestion 

Each protein solution (100 µL) was purified by polyacrylamide gel electrophoresis with a 16% polyacrylamide concentrate gel at 100 V for 10 min. The protein gel was stained with Coomassie Brilliant Blue for 2 h and then decolorized with acetic acid for 2 h. The blue bands were cut into small pieces and rinsed three times with water. Subsequently, DTT (10 mM) was added to the gel pieces at 56 °C, and the mixture was incubated for 45 min. After this, the solution was removed, and iodoacetamide (55 mM) was added to the gel. The reaction was left to proceed for 30 min at room temperature in the dark. Next, the NH_4_HCO_3_ (0.05 M)/ACN (1:1, *v*/*v*) solution (20 mL) was used to decolorize the gel, and then, the decolorizing agent was added twice every 30 min. The gel pieces were dehydrated rapidly with acetonitrile and vacuum-dried for 30 min. A trypsin/0.05 M NH_4_HCO_3_solution (1:20, *v*/*v*) was employed to digest the proteins in the gel. Each mixture was incubated at 37 °C for 18 h, and then eachsolution was transferred into a new 2-mL tube. The gel pieces were incubated at 37 °C overnight after adding ACN/H_2_O (1:1, *v*/*v*, containing 5% TFA). Subsequently, the solution was collected and dried using a vacuum centrifugal concentrator. The residue was dissolved in aqueous formic acid (0.1%) and analyzed by UPLC/Orbitrap Fusion MS/MS [60,61,62].

### 4.4. Size Exclusion Chromatography of the Digest Mixture

The molecular weight ranges of the digest mixtures were determined on a TSK G2000SWL column (7.8 mm id × 300 mm length; particle size, 5 µm) (Tosoh Bioscience, Tokyo, Japan) using a Waters 2695–2998 liquid chromatography system (Waters Instruments Co., Rochester, MN, USA). The mobile phase was comprised of a phosphate buffer (0.02 mol/L) containing sodium sulfate (0.1 mol/L). The flow rate was set to 0.5 mL/min. A 10-µL aliquot of the sample was withdrawn from the digest mixture and injected directly onto the column. The UV detection was recorded at 220 nm. The size exclusion chromatography results are displayed in Figure 1.

### 4.5. Chromatographic Separation and Mass Spectrometry

The liquid chromatography (LC) separation was conducted using a Thermo Scientific™ EASY-nLC™ 1000 HPLC system (Thermo Fisher Scientific Inc., Waltham, MA, USA). The mobile phases were composed of (A)water (with 0.1% of formic acid) and (B)acetonitrile (with 0.1% of formic acid). The peptides were loaded directly onto a homemade C18 column (75 μm id × 15 cm, 3 μm, 120 Å). The analytical mobile phase gradient was 2–6% B from 0–1 min, 6–25% B from 1–46 min, 25–35% B from 46–61 min, 35–80% B from 61–62 min, and finally 80% B for an additional 8 min. The flow rate was set to 300 nL/min for these analytical gradients. The column and autosampler were maintained at temperatures of 40 and 8 °C, respectively. The injection volume was 5 µL [63,64].

All the separated peptide fractions were analyzed using a Thermo Orbitrap Fusion^TM^ (Thermo Fisher Scientific, Waltham, MA, USA) mass spectrometer. The data were acquired at a resolution of 120,000 (@ 200 *m*/*z*) for full MS scans, followed by a high-energy-collision dissociation (HCD) fragmentation and detection of the fragment ions in the ion trap. The MS parameters were set as follows. Full Scan for MS: resolution (@ *m*/*z* 200) 120,000; scan range (*m*/*z*): 350–1550; max injection (ms): 50; and automatic gain control (AGC) target: 2.00 × 10^5^. Data-dependent MS/MS: Fragmentation HCD; NCE (%): 35; detector type: Orbitrap; AGC target: 5.00 × 10^4^; max injection (ms): 35; and dynamic exclusion (s): 60. All acquisitions and data analyses were controlled using the Progenesis QI for proteomics v3.0 (QIP) (Waters, Great Bookham, UK) and Mascot v2.5.1 (Matrix Science, London, UK) software.

LC/MS peptides mass spectrogram fingerprint method was validated under the regulation of Chinese Pharmacopoeia Commission. Seven different ions (RT 4.29 min, *m*/*z* 330.1976; RT 7.47 min, *m*/*z* 508.2743; RT 10.52 min, *m*/*z* 615.3333; RT 14.47 min, *m*/*z* 577.2941; RT 20.56 min, *m*/*z* 218.2128; RT 26.49 min, *m*/*z* 802.4413; and RT 31.95 min, *m*/*z* 246.2455) were selected for repeatability, precision, and stability, because the detected ion intensities were generated using the RT and *m*/*z* data pairs in LC/MS peptides mass spectrogram. In six mass spectrograms of the same sample solution, the RSD values of retention time and exact mass of seven ions were less than 1.0%. This suggested that the precision of method was better. In six mass spectrograms of six sample solutions prepared from the same sample, the RSD values of retention time and exact mass of seven ions were also less than 1.0%, and it revealed the repeatability of the method was better. The RSD values of retention time and exact mass of seven ions detected in 0, 2, 4, 6, 8, and 10 h were less than 1.2%, which showed the sample solution was quite stable within 10 h.

### 4.6. Multivariate Data Analyses

**Figure 5:**
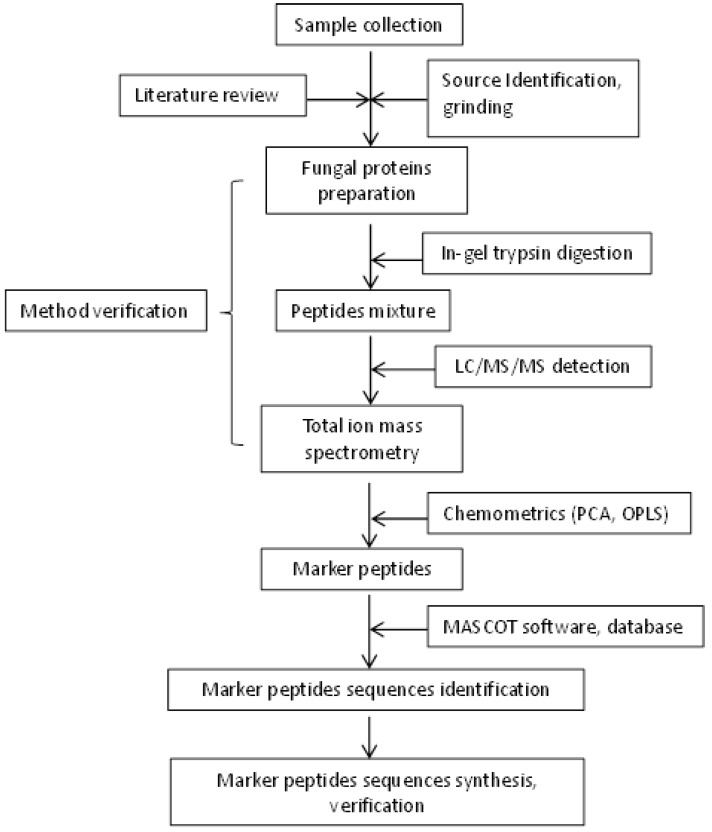
Scheme of the developed method.

Progenesis QIP was used to analyze the raw data. The following parameters were employed: retention time range: 1.0–61.0 min; detected mass range: 100–2000 Da; mass tolerance: 0.05 Da; noise elimination level: 6.00; intensity threshold: 100 counts; mass window: 0.05 amu; and retention time (RT) window: 0.2 min. No specific mass or adduct was excluded. A list of the detected peak intensities was generated using the RT and *m*/*z* data pairs. Ions in different samples were considered to be identical when they demonstrated identical RT (tolerance of 0.2 min) and *m*/*z* values (tolerance of 0.05 Da). The ion intensities for each detected peak were normalized against the sum of the peak intensities within that sample using Progenesis QIP. The resulting three-dimensional data comprising of the peak number (RT-*m*/*z* pair), sample name, and ion intensity were analyzed via unsupervised PCA. All variables were pareto-scaled prior to analysis. The scheme of the developed method was shown in Figure 5.

## 5. Conclusions

In this work, we established an efficient method that employs UPLC/Orbitrap-Fusion-MS/MS coupled with chemometrics for the identification of wild *O. sinensis* and four cultured *O.* mycelia products by identifying the marker peptide. This approach allowed for the profiling of the details of each sample so that the different marker peptides could be detected. Hence, the marker peptides could be used as powerful indexes for the identification of these mycelia and to distinguish the different mycelia in mixtures. The present approach provided a foundation for detecting the ion pairs, which came from parent ion and fragment ion of marker peptides using the MRM mode by LC/MS/MS and for developing the sensitive, stable, rapid quality control standard of the valuable Chinese medicine *O. sinensis* and its various cultured mycelia products.

## Figures and Tables

**Table 1:** The multianalyte results of the marker peptides from *Ophiocordyceps sinensis* and the four cultured *O*. mycelia.

		Marker				Mascot Matching		
Item	No.of Ions	Precursor Ion, (*m*/*z*)	Charge	Fragment Ion, (*m*/*z*)	Time, (min)	Peptide Match	Peptide Fragmentation	Protein Match
*Ophiocordyceps sinensis*	1	713.39	2		41.1	TLLEAIDSIEPPK		gi:A4U9H1
				969.52			y	(*Ophiocordyceps brunneipunctata*)
				898.48			y	
				785.4			y	
				215.13			b	
				452.25			y^0^	
				881.46			y *	
	2	851.74	3		46.2	SVEMHHEQLTEGLPGDNVGFNVK		gi: 0A060IK44
				1046.52			y	(*Ophiocordyceps sinensis*)
				949.47			y	
				497.7			b++	
				596.25			b *++	
				777.42			y	
				1208.54			b	
fermented *O. sinensis* mycelia	3	579.34	2		35	AVLSDAITLVR		gi: T5AC53
				987.58			y	(*Hirsutella sinensis*)
				874.49			y	
				787.46			y	
				672.44			y	
				488.31			y	
				142.6			b++	
	4	670.3	2		14.5	NAGSGCPTYTVGR		gi: T5AC53
				1154.52			y	*(Hirsutella sinensis)*
				1010.47			y	
				397.21			y++	
				370.13			b *	
				387.16			b	
	5	614.85	2		54.7	MVEVLGIIQAR		gi: T5AC53
				657.4			y	*(Hirsutella sinensis)*
				770.48			y	
				998.59			y	
				231.11			b	
				360.15			b	
artificial *Ophiocordyceps* mycelia	6	555.8	2		26	LESVVTSFTK		gi: 0A0B7JUZ6
				868.47			y	(*Gliocladium roseum*)
				682.37			y	
				243.13			b	
				215.12			b++	
				482.75			b++	
	7	637.34	2		44.9	HALVIYDDLSK		gi: 0A0B7JUZ6
				952.49			y	*(Gliocladium roseum)*
				740.34			y	
				577.28			y	
				209.1			b	
				534.33			b	
fermented *Ophiocordyceps* mycelia	8	849.47	1		22.6	(FAELLEK)		Unassigned
				502.32			y	
				389.23			y	
				348.15			b	
	9	799.44	2	966.52	58	YLEIIKETSNFIK	y	gi: A0A172PXZ9
				596.85			y++	(*Ophiostoma pseudocatenulatum*)
				406.19			b	
				990.55			b	
				487.26			b *++	
*O. mortierella* mycelia	10	414.75	2		13.6	(VPSSAVLR)		Unassigned
				458.3			y	
				370.24			y *	
				387.27			y	
				142.58			b++	

Note: y *(*m*/ *z*= 881.46) ion was dehydroxylated ion of y (*m*/ *z*= 898.48); b *++ (*m*/*z* = 596.25) ion was dehydroxylated ion of b (*m*/*z* = 1208.54) with two charges, b * (*m*/*z* = 370.13) ion was dehydroxylated ion of b (*m*/*z* = 387.16), b *++ (*m*/*z* = 487.26) ion was dehydroxylated ion of b (*m*/*z* = 990.55) with two charges, and y * (*m*/*z* = 370.24) ion was dehydroxylated ion of y (*m*/*z* = 387.27).

**Table 2:** The *Ophiocordyceps*-related samples’ information included in this study.

Sample Status	Claimed Names ^a^	No. of Samples	Locations
Wild fruiting body	*Ophiocordyceps sinensis*	4	Tibet
Cultured mycelium powder ^b^	Fermented *O.S.* mycelia	5	Zhejiang
	Fermented *Ophiocordyceps* mycelia	6	Jiangxi
	Artificial *Ophiocordyceps* mycelia	8	Hebei
	*O. mortierella* mycelia	5	Zhejiang

^a^ Sample names when they were collected; ^b^ cultured mycelium powder was collected from the manufacturing enterprise of each sample.
